# Advancing current approaches to disease management evaluation: capitalizing on heterogeneity to understand what works and for whom

**DOI:** 10.1186/1471-2288-13-40

**Published:** 2013-03-14

**Authors:** Arianne MJ Elissen, John L Adams, Marieke Spreeuwenberg, Inge GP Duimel-Peeters, Cor Spreeuwenberg, Ariel Linden, Hubertus JM Vrijhoef

**Affiliations:** 1 Department of Health Services Research, CAPHRI School for Public Health and Primary Care, MaastrichtUniversity, Duboisdomein 30, PO Box 616 6200MD, Maastricht, the Netherlands; 2 Department of Research and Evaluation, Kaiser Permanente Center for Effectiveness and Safety Research, Pasadena, CA, USA; 3 Department of General Practice, CAPHRI School for Public Health and Primary Care, Maastricht University, Maastricht, the Netherlands; 4 Department of Patient and Care, Maastricht University Medical Centre, Maastricht, the Netherlands; 5 Linden Consulting Group, Ann Arbor, MI, USA; 6 Department of Health Management and Policy, School of Public Health, University of Michigan, Ann Arbor, MI, USA; 7 TRANZO Scientific Centre for Care and Welfare, Tilburg University, Tilburg, the Netherlands; 8 Saw Swee Hock School of Public Health, National University of Singapore, Singapore, Singapore

**Keywords:** Chronic disease management, Quality measurement, Evaluation methodology, Multilevel regression methods, Statistical heterogeneity

## Abstract

**Background:**

Evaluating large-scale disease management interventions implemented in actual health care settings is a complex undertaking for which universally accepted methods do not exist. Fundamental issues, such as a lack of control patients and limited generalizability, hamper the use of the ‘gold-standard’ randomized controlled trial, while methodological shortcomings restrict the value of observational designs. Advancing methods for disease management evaluation in practice is pivotal to learn more about the impact of population-wide approaches. Methods must account for the presence of heterogeneity in effects, which necessitates a more granular assessment of outcomes.

**Methods:**

This paper introduces multilevel regression methods as valuable techniques to evaluate ‘real-world’ disease management approaches in a manner that produces meaningful findings for everyday practice. In a worked example, these methods are applied to retrospectively gathered routine health care data covering a cohort of 105,056 diabetes patients who receive disease management for type 2 diabetes mellitus in the Netherlands. Multivariable, multilevel regression models are fitted to identify trends in clinical outcomes and correct for differences in characteristics of patients (age, disease duration, health status, diabetes complications, smoking status) and the intervention (measurement frequency and range, length of follow-up).

**Results:**

After a median one year follow-up, the Dutch disease management approach was associated with small average improvements in systolic blood pressure and low-density lipoprotein, while a slight deterioration occurred in glycated hemoglobin. Differential findings suggest that patients with poorly controlled diabetes tend to benefit most from disease management in terms of improved clinical measures. Additionally, a greater measurement frequency was associated with better outcomes, while longer length of follow-up was accompanied by less positive results.

**Conclusions:**

Despite concerted efforts to adjust for potential sources of confounding and bias, there ultimately are limits to the validity and reliability of findings from uncontrolled research based on routine intervention data. While our findings are supported by previous randomized research in other settings, the trends in outcome measures presented here may have alternative explanations. Further practice-based research, perhaps using historical data to retrospectively construct a control group, is necessary to confirm results and learn more about the impact of population-wide disease management.

## Background

Disease management is commonly defined as a ‘system of coordinated health care interventions and communications for populations with conditions in which patient self-care efforts are significant’ [1]. Originally developed in the US, disease management interventions have been introduced in many countries to address widespread deficiencies in the care for chronically ill patients, including fragmentation, insufficient evidence-based practice, and limited self-management support [2]. However, especially outside of the US, available evidence about the impact of disease management remains uncertain and tends to be based on mostly small studies, which frequently target high-risk patients and are performed in academic settings [3]. Although some large-scale, realistic evaluations have already been conducted [4], there remains a need for better insight into the effects of comprehensive, population-based approaches, such as have been implemented in, for example, Germany and the Netherlands [5].

An important reason for this limited evidence base is the lack of universally accepted methods for ‘real-world’ disease management evaluation that are both scientifically sound and operationally feasible [6,7]. According to Linden et al. [8] three fundamental limitations preclude use of the ‘gold-standard’ randomized controlled trial (RCT). First, from a practical perspective, population-wide implementation of approaches can make it difficult to find a suitable number of control subjects. Second, withholding treatment that is assumed to be effective from control patients poses an ethical dilemma. Third and most important, however, the strict in- and exclusion criteria limit generalizability of findings across patients and contexts. Observational research designs are more suitable for practice-based disease management evaluation yet commonly have methodological flaws that limit the validity and reliability of findings [9].

Advancing existing methods for disease management evaluation in routine situations where randomization is not possible will be pivotal in drawing valid conclusions about the impact of this care concept on the quality and outcomes of chronic care provision. Evaluation methods must account for the presence of heterogeneity in effects of disease management, produced by differences in interventions and targeted patients [10-13]. This variation necessitates calculation of more detailed effect estimates than the commonly assessed ‘grand means’ across large populations of patients, if they are to be informative for day-to-day clinical practice.

The aim of this paper is to introduce multilevel regression methods as useful techniques for the analysis of patient data in practice-based disease management evaluation. These methods enable researchers to identify differences in outcomes as a function of features of the intervention and/or patient population, and, in so doing, support efforts to create effective and efficient disease management strategies. The article starts with a brief, non-technical description of the proposed analytic approach. Subsequently, a worked example is given of its application in the evaluation of a population-wide disease management intervention for type 2 diabetes mellitus implemented in the Netherlands. This evaluation, which was part of the European collaborative DISMEVAL (‘Developing and Validating Disease Management Evaluation Methods for European Health Care Systems’) project [5,14], was designed as an uncontrolled cohort study using routine patient data gathered retrospectively from clinical practice.

### Multilevel regression methods: what and why?

In health research, especially studies conducted in practice settings, data commonly have a hierarchical nature, with variable measures – such as cholesterol measurements – clustered within different levels of the hierarchy [15]. For example, in a practice-based study examining factors that influence the use of shared-decision making in general practice, patients would be clustered within physicians, who in turn might be nested within group practices. Traditional statistical methods, such as linear regression analysis, tend to ignore the multilevel structure of routine health data and do not account for the possibility of similarities among individuals clustered within higher-level units [16]. Yet in reality, subjects within clusters are often more alike than randomly chosen individuals with regard to important characteristics, such as sociodemographic features. Hence, assuming that observations within clusters are uncorrelated is not realistic and can result in false conclusions about associations between particular variables [16,17].

Multilevel regression methods enable researchers to explicitly include the hierarchical nature of practice data into their analyses [15]. Similar in essence to simple regressions, multilevel regression entails predicting an outcome variable according to the values of one or more explanatory variables, which may be measured at different levels in the hierarchy [18]. The latter are usually called covariates, i.e. characteristics that might influence the size of a particular intervention’s effects. Person-level covariates can enter the model in two different ways. First, they may appear as ordinary covariates at level one of the hierarchy. Second, they may appear in interaction terms with intervention characteristics. These interaction terms capture the idea of ‘effect modification’ by allowing the person-level variables to modify the intervention effects.

Applying multilevel regression methods is of particular relevance when patient outcomes are regarded as heterogeneous, as is typically the case with disease management. In a simple two-level model, total heterogeneity in effects can be divided into two variance components: within-groups and between-groups [16]. Multilevel regression techniques make it possible to capitalize on this variation in three ways, the outcomes of which can support further improvements in the quality and outcomes of disease management [19]. First, it enables identification of subgroups of patients for whom treatment is associated with the most positive effects. Second, it permits investigation of characteristics of an intervention, either active (treatment features) or passive (setting features), that are associated with favorable outcomes [18,20]. Third, it allows for multiple factors measured at different levels in the hierarchy to be examined together, the results of which may facilitate stratified medicine. In the remainder of this paper, we will show how multilevel regression methods were applied in our evaluation of the Dutch approach to disease management for type 2 diabetes.

### Worked example: Dutch disease management evaluation

In 2007, the Netherlands Organization for Health Research and Development (ZonMw) started a governmentally subsidized pilot called the ‘Integrated Diabetes Care research program’ to overcome existing barriers to coordination of care for type 2 diabetes patients. As part of the pilot, ten so-called ‘care groups’ – i.e. provider networks in primary care, gathering mostly general practitioners (GPs) and affiliated personnel – were offered financial incentives to start experimenting with a bundled payment system that allows the different components of outpatient care for type 2 diabetes to be purchased, delivered, and billed as a single product (i.e. a disease management intervention) [21,22]. Care groups are responsible for all patients covered by their care program; they deliver services themselves and/or subcontract services from other providers, such as physical therapists, dietitians, laboratories, and, to a limited extent, specialists [23]. A national evidence-based care standard for type 2 diabetes care guides negotiations between care groups and health insurers on the content and price of diabetes care programs [24].

One of the main goals of implementing the bundled payment system was to stimulate the transfer of non-complex chronic care from the hospital setting to general practice, which traditionally is a strong sector in the Netherlands and widely regarded as most suitable to serve as ‘medical home’ for chronically ill patients [25]. Nearly all Dutch citizens are registered with a GP, who constitutes the first point of contact for care-seeking individuals and acts as gatekeeper for secondary care [23]. Although some regional bundled payment contracts include a limited amount of specialist care, these services are generally reserved for patients with complex and unstable long-term health problems, such as type 1 diabetes patients and/or multimorbid patients.

Despite uncertainty about the effectiveness of the new financing and delivery system, care groups with bundled payment contracts for type 2 diabetes disease management interventions rapidly achieved national coverage in the Netherlands [26]. For evaluators, this broad dispersion, combined with the unsuitability of using historic controls – evidence suggests that the quality of diabetes care improves over time as a secular trend [27] – limits the use of experimental comparisons. Thus, to analyze the impact of the Dutch approach to disease management for type 2 diabetes, we conducted an uncontrolled, practice-based cohort study using multilevel regression methods. Although these methods precluded the establishment of cause-effect relationships, they enabled us to identify trends in outcome measures that might suggest that components of the intervention under consideration have an effect for (subgroups of) type 2 diabetes patients [28]. Our study was conducted in five steps: (1) participant selection, (2) data collection and validation, (3) variable definition, (4) data analysis, (5) outcome interpretation.

## Methods

### Participant selection

We selected a convenience sample of 18 care groups, which were set up between the years 2006 and 2009. Nine groups were part of the pilot of the bundled payment system, for which they were selected ensuring diversity in geographical location and size [21]. We used the same criteria to include nine additional, non-experimental groups, i.e. regional initiatives that have a bundled payment contract for diabetes disease management interventions with a health insurer but do not receive (financial) support from the pilot. The 18 care groups represent all but one region of the Netherlands, employ between 7 and 230 GPs per group, and cover patient populations ranging from 348 to 18,531 persons. From each group, we selected all type 2 diabetes patients with at least one registered visit to general practice during the research period (N = 106,623), which – depending on the availability of data – was either 20 or 24 months between January, 2008 and December, 2010. We excluded type 1 diabetes patients (N = 1567), because they are treated primarily by specialists.

### Data collection and validation

The bundled payment system for chronic care in the Netherlands requires care groups to register a specific number of performance indicators for care processes and clinical outcomes on an annual basis. We retrospectively gathered patient data on a selection of those indicators from the clinical information systems of our 18 care groups. Data plausibility was verified through range checks, we removed outliers in clinical values based on cut-off points determined by Dutch diabetes experts (see Table 1). Missing values were not imputed.

**Table 1:** Cut-off points for clinical outcome data

**Indicator**	**Lower**	**Upper**	**Excluded (N)**	**Excluded (%)**
Glycated hemoglobin (HbA1c) (mmol/mol)	18	108	913	0.51
LDL cholesterol (mmol/l)	1	7.3	2110	1.31
Systolic blood pressure (mmHg)	70	250	25	0.01
BMI (kg/m^2^)	16	70	123	0.08

Because patient data were not available for the period before introduction of the bundled payment system, we used the last measurement of each clinical outcome registered per patient during the first year of the research period (or first eight months, for two groups with a 20-month research period) as baseline. Thus, the baseline data used in this study represent data at the introduction of the disease management intervention (i.e. bundled payment system). Given that patients were enrolled at different time points during the first year, using the last measurement registered in that period as baseline was preferred over the first measurement to minimize heterogeneity in follow-up duration between patients. This is a conservative decision because for some cases a portion of the program effects will be incorporated in the baseline measurements.

**Figure 1:**
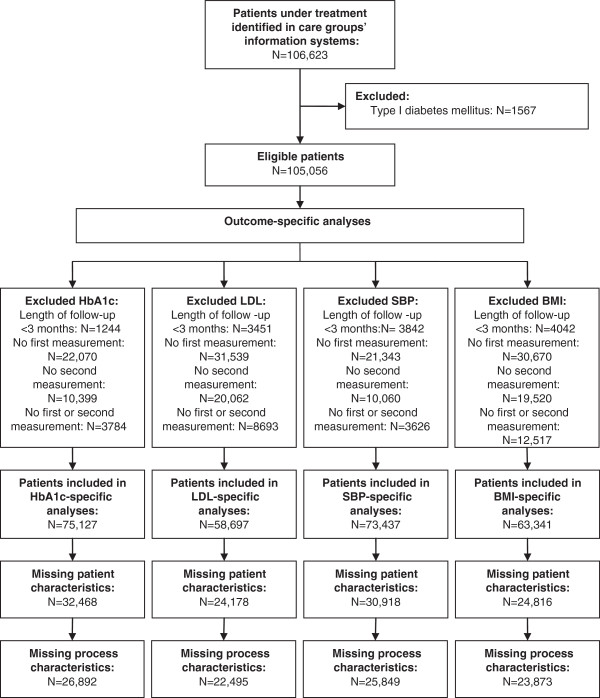
Study flow chart.

To identify trends in outcome measures, we calculated changes in clinical parameters from baseline to follow-up, which was operationalized as the last measurement of each clinical outcome per patient registered during the second year of the research period. Large correlations between observations within person make the choice of modeling change scores rather than separate cross-sections compelling for maximizing statistical power. Modeling change scores also controls for unmeasured but fixed person-level covariates. Before conducting each outcome-specific analysis, we excluded patients who: (1) lacked valid registrations of baseline or follow-up measurement, or both, (2) missed registrations of one or more of the characteristics used as covariates in the multilevel regression analyses, and/or (3) had an observation period between baseline and follow-up of less than three months. The maximum length of follow-up per patient was 23 months. The study flowchart is shown in Figure 1.

### Variable definition

**Figure 2:**
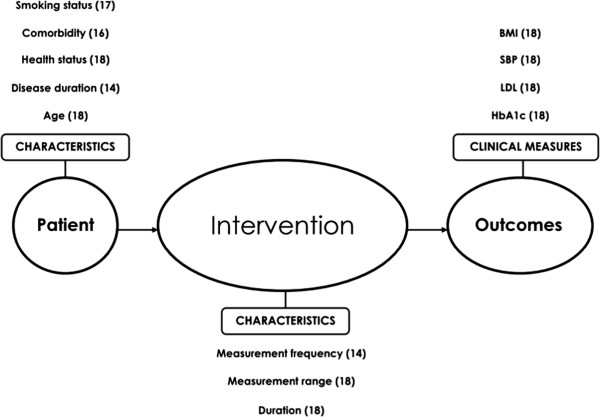
Overview of research variables (and registration in number of care groups).

To enable investigation of heterogeneity in effects on clinical outcomes, we defined relevant variables relating to patient characteristics and active features of the intervention. Figure 2 shows a graphical conceptualization of the included variables and the number of care groups able to provide data on those variables.

With regard to intervention features, we coded measurement frequency as the number of registrations of each clinical outcome during follow-up. To describe measurement range, we assessed the amount of different outcomes registered per patient over baseline, which could be a maximum of eight (i.e., glycated hemoglobin, total cholesterol, low- and high-density lipoprotein, triglycerides, systolic and diastolic blood pressure, and body mass index). Duration of care was defined as an individual patient’s length of follow-up in months. To describe patients, we used these characteristics: age (in years), disease duration (in years), health status, diabetes complications, and smoking status. Health status was determined by the baseline values of each clinical outcome. Diabetes complications, registered since diagnosis of type 2 diabetes (that is, either before or during the research period), could comprise one or more of the four most frequently registered co-occurring conditions across the included care groups, i.e. angina pectoris, myocardial infarction, stroke, and/or transient ischemic attack. We dichotomized smoking status as previous or non-smoker versus current smoker. Finally, we defined clinical outcomes as changes over baseline in glycated hemoglobin (HbA1c), low-density lipoprotein (LDL), systolic blood pressure (SBP), and body mass index (BMI).

### Data analysis

We conducted univariate analyses to describe patient and intervention characteristics, which were reported either as means and associated standard deviations (age, disease duration, health status), median values (measurement frequency, length of follow-up), or percentages (diabetes complications, smoking, measurement range). Using paired sample t-tests (two-sided, α = 0.05), we calculated the care group-specific and overall mean differences in clinical outcomes between baseline and follow-up, and 95% confidence intervals. To quantify the heterogeneity in clinical results among our 18 care groups, we calculated the I^2^ statistic on the basis of the chi-square (*χ*^2^) test. I^2^ describes the percentage of total variation in effects across groups that is due to heterogeneity rather than chance. The principal advantage of I^2^ – which lies between 0 and 100% with larger values showing increasing heterogeneity – is that it can be calculated and compared across groups irrespective of differences in size and type of outcome data [29].

For outcomes showing moderate (I^2^ > 50%) to high (I^2^ > 75%) heterogeneity, multivariable, two-level hierarchical regression models – with patients at level one and care groups at level two – were used to analyze the influence of selected covariates on changes in clinical outcomes between baseline and follow-up. Two separate models were fit to test all covariates related to patient and intervention characteristics, respectively. In a third series of models, we investigated every possible two-way interaction between patient characteristics and intervention features. The models used were similar to the kind that might be fit in a multi-center study, i.e. mixed models incorporating a random care group effect (PROC MIXED command in the SAS® 9.2 Software), which was considered most suitable given the possibility of ‘residual heterogeneity’ [30]. Where possible, covariates were analyzed both as continuous and as categorical variables, with categories based on scientific literature (age [31] and disease duration [32]), median values (measurement frequency and length of follow-up), or, in the case of baseline health status, on the target values for clinical parameters incorporated in the Dutch care standard for type 2 diabetes [24]. Measurement range was categorized as eight registered outcomes versus less than eight registered outcomes.

For each outcome, we calculated the intraclass correlation coefficient (ICC) which describes the proportion of total heterogeneity in effects attributable to between-group variance rather than within-group variance [33]. We examined collinearity with the variance inflation factor (VIF): a VIF value greater than 10 is generally taken as an indication of serious multi-collinearity [34]. The regression coefficients obtained from our multilevel analyses describe how a specific effect estimate changes following a unit increase in a covariate, whether there is actually a relationship between both is expressed in the statistical significance. We expressed ‘explained heterogeneity’ as the percentage change in between-group variance (τ^2^) and within-group variance (σ^2^) after correcting for selected covariates.

## Results

### Interpretation of results

#### Univariate analyses

Included in our analyses were 105,056 patients, about half of whom (50.6%) were female. The average age of the research population was 65.7 (±11.9) years and average disease duration 4.8 (±5.6) years. Further details are shown in Table 2. With regard to care processes, patients’ SBP was assessed most frequently during follow-up (median = 4), followed by BMI (median = 3), and HbA1c (median = 2). LDL was measured least often (median = 1). Across groups, the average share of patients with the maximum measurement range varied from 44.4 to 86.7%, with a mean of 62.3%. Median length of follow-up was 12 months.

**Table 2:** Characteristics of the research population

**Characteristic**	**Patients for whom characteristic is known (total =105,056)**	**Estimate**
	**% (N)**	**Mean ± SD**
Baseline age	99.9 (105,013)	65.7 ± 11.9
Baseline diabetes duration	71.9 (75,498)	4.8 ± 5.6
Baseline health status		
HbA1c (mmol/mol) [target < 53]	71.5 (75,127)	50.2 ± 9.8
LDL cholesterol (mmol/l) [target < 2.5]	55.9 (58,697)	2.6 ± 0.9
SBP (mmHg) [target < 140]	69.9 (73,437)	140.4 ± 18.0
BMI (kg/m^2^) [target < 25]	60.3 (63,341)	29.7 ± 5.2
	**% (N)**	**% (N)**
Diabetes complications^†^	94.5 (99,278)	
None		84.2 (75,357)
One or more		15.8 (14,165)
Smoking status	74.6 (78,384)	
No or Ex-smoker		81.6 (63,943)
Current smoker		18.4 (14,441)

NOTE: ^†^ Included were four major diabetes complications: angina pectoris, myocardial infarction (MI), stroke, transient ischemic attack (TIA).

Table 3 presents the mean changes over baseline in clinical outcomes across the total of 18 care groups. Overall, we found a small, non-significant increase in HbA1c levels between baseline and follow-up, while small but significant reductions in mean levels were observed for LDL and SBP. Except for BMI, all outcomes showed moderate to high statistical heterogeneity, from 57% for SBP to 98% for HbA1c, suggesting that the effects of the diabetes disease management interventions on these outcomes were inconsistent across care groups. To elucidate this heterogeneity and identify trends in the measured results, multilevel regression analyses were conducted.

**Table 3:** Results of the univariate analyses per clinical outcome

**Clinical outcome**	**Care groups (N)**	**Patients (N)**	**Mean difference [95% CI]**	**Heterogeneity (I**^**2**^**)**
HbA1c (mmol/mol)	18	75,127	0.17 [−0.60, 0.93]	98%*
LDL (mmol/l)	18	58,697	−0.09 [−0.13, -0.05]*	93%*
SBP (mmHg)	18	73,437	−0.95 [−1.25, -0.64]*	57%*
BMI (kg/m^2^)	18	63,341	−0.04 [−0.10, 0.02]	0%

NOTE: * Statistically significant (p < 0.05).

#### Multilevel regression analyses

The results of the multilevel regression analyses are summarized in Table 4, which shows the changes in between- and within-group heterogeneity in effects on HbA1c, LDL and SBP, after correcting for included covariates, with the direction of covariate influence indicated (positive or negative). We observed that the vast majority of variance in the effects of disease management on clinical outcomes occurred within care groups rather than between groups, with ICCs ranging from 0.1 to 4.3% across outcomes. Simultaneously correcting for known patient characteristics resulted in the most considerable reductions in within-group variance in effects. We found no evidence of multi-collinearity in any of the regression models.

**Table 4:** **Effect of active intervention features and patient characteristics on changes in HbA1c, LDL and SBP over baseline and associated changes in between-group(τ**^**2**^**) and within-group(σ**^**2**^**) variance in effects**

	**HbA1c**			**LDL**			**SBP**		
	**N**	**Mean difference [95% CI]**	**RC**	**N**	**Mean difference [95% CI]**	**RC**	**N**	**Mean difference [95% CI]**	**RC**
Active intervention features									
*Measurement frequency*			-			-*			-*
≤Median	27,322	0.09 [−0.85, 1.03]		25,420	−0.08* [−0.12, -0.05]		21,764	−0.99* [−1.57, -0.41]	
>Median	20,913	−0.06 [−1.47, 1.36]		10,782	−0.20* [−0.26, -0.15]		25,824	−1.01* [−1.38, -0.64]	
*Measurement range*			-*			-			+*
<8 outcomes	15,641	0.13 [−1.08, 1.34]		6500	−0.10* [−0.15, -0.05]		14,397	−1.60* [−2.02, -1.17]	
8 outcomes	51,820	0.05 [−0.66, 0.75]		45,301	−0.10* [−0.14, -0.06]		50,392	−0.79* [−1.15, -0.43]	
*Length of follow-up*			+*			+*			+*
≤1 year	57,069	0.02 [−0.77, 0.81]		43,901	−0.09* [−0.13, -0.05]		55,686	−1.27* [−1.60, -0.95]	
>1 year	18,058	0.53 [−0.22, 1.27]		14,796	−0.11* [−0.15, -0.06]		17,751	−0.04 [−0.52, 0.44]	
Change in *τ*^*2*^		26.0%			−37.0%			15.6%	
Change in *σ*^*2*^		−0.1%			0.7%			5.2%	
Patient characteristics									
*Age*			-*			+*			+*
≤59	20,538	0.21 [−0.56, 0.98]		15,857	−0.12* [−0.16, -0.07]		20,139	−0.57* [−0.92, -0.23]	
60-69	24,204	0.23 [−0.55, 1.02]		19,364	−0.09* [−0.13, -0.05]		23,689	−1.00* [−1.38, -0.62]	
≥70	30,382	0.03 [−0.78, 0.83]		23,474	−0.08* [−0.12, -0.03]		29,605	−1.34* [−1.72, -0.96]	
*Disease duration*			+*			-			+
≤2	21,261	-0.12 [−1.12, 0.88]		16,756	−0.14* [−0.19, -0.08]		21,673	−1.04* [−1.47, -0.62]	
3-5	13,342	0.11 [−0.76, 0.98]		10,607	−0.08* [−0.12, -0.03]		13,117	−0.55* [−0.97, -0.13]	
≥6	20,474	−0.07 [−1.08, 0.95]		15,857	−0.06* [−0.10, -0.01]		19,645	−1.22* [−1.61, -0.84]	
*Baseline health*			-*			-*			-*
Good	51,545	1.79* [1.17, 2.41]		29,311	0.15* [0.12, 0.18]		42,784	4.59 [4.21, 4.97]	
Moderate	21,637	−2.62* [−3.46, -1.78]		19,984	−0.17* [−0.20, -0.14]				
Poor	1945	−16.82* [−18.7,-15.0]		9402	−0.72* [−0.77, -0.67]		30,653	−8.91 [−9.67, -8.16]	
*Diabetes complications*			+*			-*			-*
No	53,065	0.04 [−0.63, 0.71]		39,721	−0.10* [−0.14, -0.06]		50,780	−0.93* [−1.29, -0.58]	
Yes	10,183	−0.06 [−0.76, 0.65]		7994	−0.08* [−0.15, -0.01]		9788	−1.25* [−1.75, -0.75]	
*Smoking status*			+*			-			-
No/previously	46,277	0.23 [−0.28, 0.73]		38,294	−0.09* [−0.13, -0.04]		46,908	−0.90* [−1.24, -0.56]	
Yes	10,375	0.20 [−0.85, 1.25]		7762	−0.11* [−0.15, -0.06]		10,504	−1.07* [−1.54, -0.59]	
Change in *τ*^*2*^		−12.5%			−33.9%			74.8%	
Change in *σ*^*2*^		−23.5%			−21.7%			−29.9%	

NOTE: N indicates number of patients, CI, confidence interval, RC, regression coefficient, +, positive regression slope, -, negative regression slope, *statistically significant (p < 0.05), baseline health status is defined according to the target values of the national diabetes care standard: Good (HbA1c ≤ 53 mmol/mol, LDL < 2.5 mmol/l, SBP ≤ 140 mmHg), Moderate (HbA1c 54-74 mmol/mol, LDL 2.5-3.5 mmol/l), Poor (HbA1c ≥ 75 mmol/mol, LDL > 3.5 mmol/l, SBP > 140 mmHg).

The multilevel regression model incorporating intervention characteristics showed that two covariates significantly influenced the effects of disease management in a consistent manner across clinical outcomes. Whereas a greater measurement frequency of clinical outcomes was associated with better results on those outcomes, longer length of follow-up was accompanied by diminishing positive effects on HbA1c, LDL and SBP. The results for measurement range were inconsistent across clinical outcomes.

**Figure 3:**
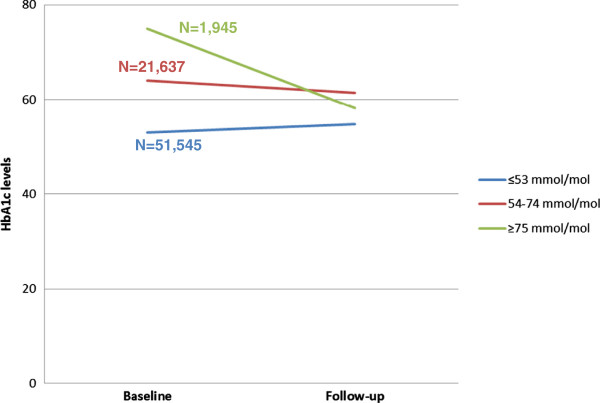
Glycemic control (mmol/mol) from baseline to follow-up according to the target values of the Dutch care standard for type 2 diabetes mellitus.

The model for patient characteristics found significant and consistent associations between baseline clinical values and intervention effects, suggesting that the impact of disease management becomes progressively better as patients’ initial health values are poorer. Figure 3 depicts how across the 18 care groups, patients with a baseline HbA1c ≥75 mmol/mol achieved a mean reduction in this clinical measure of 16.8 mmol/mol (95% CI: -18.7, -15.0), whereas those starting within the target range for HbA1c (≤53 mmol/mol) experienced a slight deterioration in glycemic control (1.79 mmol/mol [95% CI: 1.2, 2.4]). The HbA1c levels of those with baseline values between 54 and 74 mmol/mol reduced by an average of 2.6 mmol/mol (95% CI: -3.5, -1.8). For SBP and LDL, similar trends were found. Those with poor baseline values tended to show the greatest improvements. The findings for age, disease duration, diabetes complications and smoking status were less conclusive and inconsistent across clinical outcomes.

The multilevel regression models incorporating covariates related to both patients and the intervention found one significant two-way interaction that was consistent across all included outcomes. Thus, for patients with poorer initial values of a particular clinical outcome, more frequent assessment of that outcome was associated with progressively greater health improvements than was the case for patients with healthier baseline levels.

## Discussion

Evaluating the effects of population-wide disease management interventions implemented in actual health care settings is a complex undertaking [35]. The Dutch example described in this paper illustrates how practical issues, such as a lack of suitable control patients, can limit the use of experimental comparisons to establish whether a given intervention yields a ‘true’ effect. Indeed, attributing observed changes in variable measures to the disease management approach under consideration is one of the key challenges in practice-based evaluation [5,14]. In cases like ours, where rigorous performance assessment is complicated because data collection is tied to the intervention and real baseline data is lacking, a frequently used solution is to report data from a first observation period as baseline and to use changes from this baseline as estimates of effects [6]. Such an observational approach is susceptible to various sources of confounding and bias, which threaten the internal validity of study results and cannot always be observed and/or measured so as to enable statistical adjustment. In evaluating complex health service innovations such as disease management, however, even randomization is unlikely to successfully control for the large number of factors and interactions on different levels that might influence outcomes [36].

Although results must be interpreted with caution, given the methodological limitations of uncontrolled research, the value of our proposed methods lies in the opportunity to analyze routine data from clinical practice in a manner that produces meaningful results for further development of disease management strategies. Rather than providing a single effect estimate across many patients, which offers little guidance on what works and for whom, multilevel regression models allow researchers to capitalize on existing heterogeneity in effects by conducting a more granular assessment of the impact of an intervention’s features on the health outcomes of different patient groups. Our univariate analysis results demonstrate that a simple, unclustered comparison of Dutch disease management patients’ baseline and follow-up clinical measures would have led to the conclusion that the effects of the intervention are small at best. Yet our multilevel regression findings reveal that for patients with poor baseline clinical values, disease management was associated with significant and clinically relevant health improvements after a median follow-up of 12 months. Although this might suggest regression to the mean, which is a common phenomenon in disease management research, this is to some extent refuted by the small percentage of patients (17% for HbA1c) in the healthiest disease categories whose clinical values moved towards to the mean, despite the degenerative nature of diabetes. A 2008 large-scale, practice-based disease management evaluation conducted in Germany [4] as well as a recent meta-analysis of 41 RCTs [10] also found that disease management is most beneficial for poorly controlled diabetes patients, which – given that the vast majority of our patients had healthy baseline values of most clinical parameters – provides a plausible explanation for the small average effects of the Dutch disease management strategy for type 2 diabetes on health outcomes.

With regard to the effectiveness of different intervention features, our covariate analyses suggest that particularly for patients with poor disease control, intensive monitoring of clinical values might be an important intervention feature that is associated with better health outcomes. Other studies of disease management for diabetes have shown a similar association between more intensive interventions and better glycemic control [10,37]. The well-known population management model used by Kaiser Permanente divides patients with chronic conditions into three distinct groups based on their degree of need: (1) supported self-management, for patients with a relatively low level of need for health care (65-80%), (2) disease management, for patients at increased risk because their condition is unstable (15-30%), and (3) case management, for highly complex patients requiring active management by specialists (5%), such as type 1 diabetes patients in the Netherlands [38,39]. Further research is necessary to assess whether intensive disease management might indeed be redundant for the relatively healthy subgroup of diabetes patients and could be substituted by adequate self-management support programs that integrate primary care and community services [40]. Future studies might also investigate the impact of passive intervention characteristics (i.e. setting features) on changes in patients’ health outcomes. While a separate, unreported analysis of four passive intervention characteristics in this research – that is, experimental status of the care groups (pilot vs. non-pilot), care group size, diabetes care bundle price, and level of collaboration with specialists – demonstrated no significance for any of the studied outcomes, other factors could be of more relevance [5].

Also in line with previous research, we found that longer length of follow-up was accompanied by less positive effects on clinical outcomes [10,11]. Although this seems counterintuitive, given that increased measurement frequency was accompanied by better results, there is no dose–response relationship in the Dutch disease management approach, which means that patients with a longer observation period were not necessarily seen more often than patients followed over a shorter time frame. A plausible explanation for the identified association between length of follow-up and clinical outcomes could be that the positive effects of education on patients’ self-management behavior – and, consequently, their glycemic control – are difficult to maintain over time, which means that effects measured after a short duration of care might be overestimated [41,42].

### Limitations

Although our findings are confirmed by previous randomized research, the trends in outcome measures presented here may have alternative explanations that cannot be explored within the available data. A cautious approach would therefore be to treat these results as exploratory and look for further opportunities to confirm them in other settings, perhaps using historical benchmarking data derived from a comparable population (matched within strata) and corrected for secular trends. In particular the counter-intuitive association between length of follow-up and clinical outcomes might be explained by some unmeasured confounders, such as patients’ socioeconomic status or educational level, both of which are known to greatly influence individuals’ health behavior [43]. Alternatively, the lack of pre-intervention data may have introduced post-treatment bias, which leads to underestimation of intervention effects and could also to some extent explain results not lasting over time. Future research would benefit from analyzing multiple repeated measurements over time, the opportunity for which was limited in this study due to the recent implementation of the studied disease management strategy in the Netherlands.

Bias might also have been introduced by missing values, which were numerous in the routine data provided by our 18 care groups and necessitated exclusion of 28 to 44% of patients across the four outcome-specific analyses. Nonetheless, our findings cover a relatively large population (approximately 14% of known diabetes patients in the Netherlands in 2011 [44]), which did not differ from other diabetes populations studied in the Netherlands in terms of average age and disease duration, nor was the percentage of smokers different from that in the overall Dutch population [21,45,46]. The prevalence of diabetes complications, however, was considerably lower in our research group as compared to the total population of Dutch diabetes patients [47]. This observation might signify registration problems but could also indicate that patients with co-occurring conditions are more likely to be treated by specialists than by primary care providers.

## Conclusions

Despite concerted efforts to adjust for potential sources of confounding and bias, there ultimately are limits to the validity and reliability of findings from uncontrolled research based on routine intervention data. While our findings are supported by previous randomized research in other settings, the trends in outcome measures presented here may have alternative explanations. Further practice-based research, perhaps using historical data to retrospectively construct a control group, is necessary to confirm results and learn more about the impact of population-wide disease management.

## Competing interests

The authors declare that they have no competing interests.

## Authors’ contributions

AE participated in study design, data acquisition, analysis and interpretation, and drafted the manuscript. ID, CS, and HV were involved in study design, data acquisition, analysis and interpretation, and helped to critically revise the manuscript. MS, JA, and AL were involved in study design and analysis, and helped to critically revise the manuscript. All authors read and approved the final manuscript.

## Pre-publication history

The pre-publication history for this paper can be accessed here:

http://www.biomedcentral.com/1471-2288/13/40/prepub
